# Craniofacial, dental, and molecular features of Pyle disease in a South African child

**DOI:** 10.1038/s41405-022-00120-w

**Published:** 2022-09-22

**Authors:** Manogari Chetty, Imaan Roomaney, Chandré Oosterwyk, Noluthando Manyisa, Christian Domilongo Bope, Gloudi Agenbag, Ambroise Wonkam

**Affiliations:** 1 Department of Craniofacial Biology, Faculty of Dentistry, University of Western Cape, Bellville, South Africa; 2 Department of Pathology, University of Cape Town, Cape Town, South Africa; 3 Divsion of Human Genetics, Department of Medicine, Faculty of Health Sciences, University of Cape Town, Cape Town, South Africa; 4 University of Kinshasa, Kinshasa, Democratic Republic of Congo; 5 Centre for Bioinformatics, Department of Informatics, University of Oslo, Oslo, Norway; 6 Department of Genetic Medicine, Johns Hopkins University School of Medicine, Baltimore, USA

**Keywords:** Diseases, Special care dentistry

## Abstract

**Introduction:**

Pyle Disease (PD), or familial metaphyseal dysplasia [OMIM 265900], is a rare autosomal recessive condition leading to widened metaphyses of long bones and cortical bone thinning and genu valgum. We detail the oro-dental and molecular findings in a South African patient with PD.

**Methods:**

The patient underwent clinical, radiographic and molecular examinations. An exfoliated tooth was analysed using scanning electron microscopy and was compared to a control tooth.

**Results:**

The patient presented with marked Erlenmeyer-flask deformity (EFD) of the long bones and several Wormian bones. His dental development was delayed by approximately three years. The permanent molars were mesotaurodontic. The bones, including the jaws and cervical vertebrae, showed osteoporotic changes. The lamina dura was absent, and the neck of the condyle lacked normal constrictions. Ionic component analysis of the primary incisors found an absence of magnesium. Sanger sequencing revealed a novel putative pathogenic variant in intron 5 of *SFRP4 (*c.855+4delAGTA) in a homozygous state.

**Conclusion:**

This study has reported for the first time the implication of a mutation in the *SFRP4* gene in an African patient presenting with PD and highlights the need for dental practitioners to be made aware of the features and management implications of PD.

## Introduction

Pyle disease (PD) [OMIM 265900] was first described by Edwin Pyle, an orthopaedic surgeon in the United States of America, in 1931 [1]. He described a five-year-old boy who had knock-knees (genu valgum) and under-modelling of the metaphyses of his tubular bones [1, 2]. The disorder was subsequently named familial metaphyseal dysplasia or Pyle Disease [3]. PD is a rare condition with less than 30 cases reported in the literature to date [4–6].

**Fig. 1:**
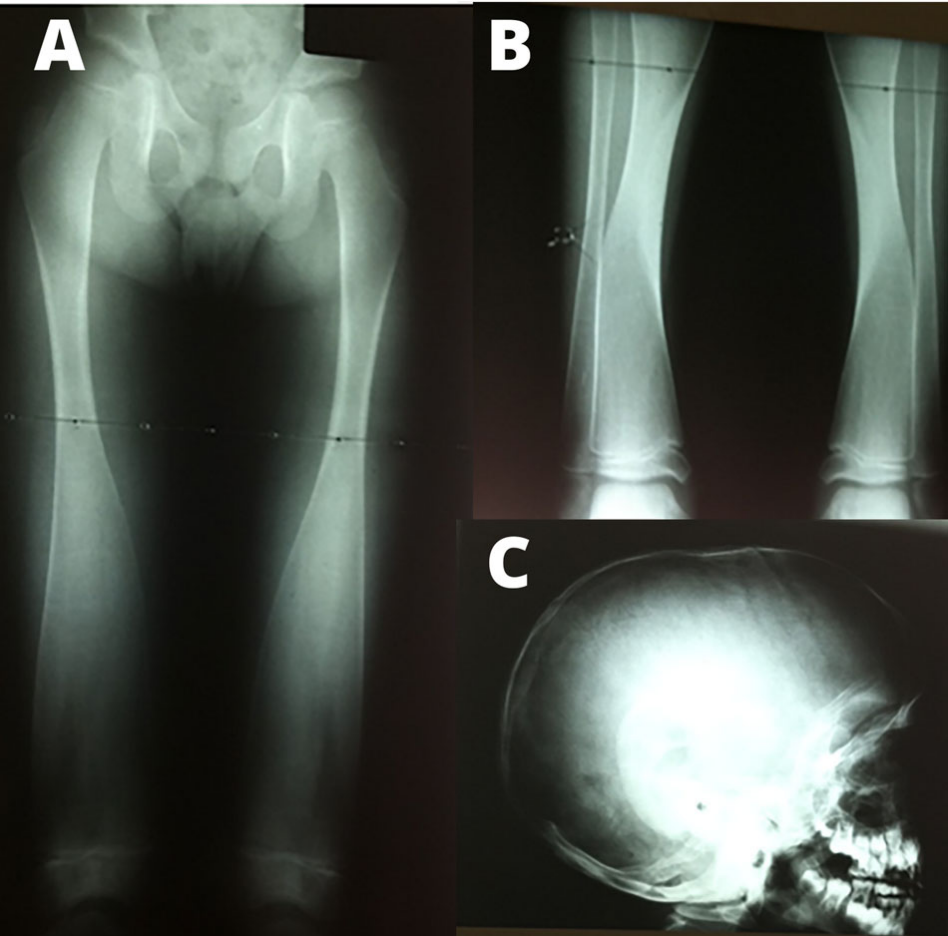
Radiographic images of a 10-year-old male with Pyle disease. **A**, **B** Erlenmeyer Flask deformity of the long bones. **C** Calvaria with Wormian bones.

The radiographic features of PD include long bones with expanded trabecular metaphyses, a thin cortical plate, and fragile bones prone to fractures that go through the wide metaphyses [2, 5, 7]. This widening of the distal aspects of long bones is known as Erlenmeyer-flask deformity (EFD) (Fig. 1A, B) [6]. In EFD, the dimetaphyseal region has a straight border with cortical thinning resulting in an Erlenmeyer-flask like shape [8]. Although EFD has been found in 20 rare conditions, it is particularly striking in PD, affecting all the long bones [8]. Additionally, variable sclerosis of the cranial bones is also a feature of PD (Fig. 1C). These features are very similar to that of Craniometaphyseal Dysplasia (CMD); however, a lack of cranial nerve compression and milder skull involvement in PD differentiates the conditions [8, 9].

PD is autosomal recessive, with several affected families identified in the literature [2, 9, 10]. Recently the *secreted frizzled-related protein* 4 (sFRP4), (606570) on chromosome 7p14 has been identified as a causative gene [7, 11, 12]. Using knock-out mice, Simsek et al. (2016) showed that the *SFRP4* gene is associated with regulation of WNT and BMP signalling. The reports by Simsek et al. (2016) and Galada et al. (2017), identified mutations in the *SFRP4* gene in four unrelated families from India, Japan and Turkey as the causative factors resulting in PD. All four reported mutations result in a truncated protein, which may activate nonsense-mediated mRNA decay, or produce a transcribed but non-functional protein [6]. The *SFRP4* gene is encoded by six exons and synthesises a protein that can bind to the WNT ligands and initiate antagonism of the WNT/frizzled pathway [13]. In addition, deletion of the *SFRP4* gene leads to increased trabecular bone mass and affects proper cortical bone thickness and strength [7].

Due to the condition’s rarity, there are very few studies evaluating the oro-dental features of PD. The most frequently described features have been prognathism, dental caries and malocclusion [2, 5, 14, 15]. Authors have also identified multiple retained deciduous teeth, unerupted permanent teeth, taurodontism, and discontinuation of the mandible’s radiographic labial cortex in a PD patient [15]. In this report, we detail the oro-dental findings of a South African male. The non-dental features of the patient at seven years were previously described by Wonkam et al. (2016). Additionally, we explore the mutations in the *SFRP4* gene that has not been studied in patients of African ancestry.

### Case report

A male of Cape Mixed Ancestry, aged ten years, presented to the University of Cape Town Genetics Clinic at Red Cross Children’s Hospital. He had suffered multiple fractures of his wrist and fingers following minor trauma. Radiographical studies of the patient were diagnostic of PD. He presented with marked EFD of the long bones (Fig. 1A, B). The skull had several Wormian bones with a flat forehead and a thin calvarium (Fig. 1C). On a recall visit, he was referred to the University of Western Cape, Faculty of Dentistry, to evaluate his oral health status.

**Fig. 2:**
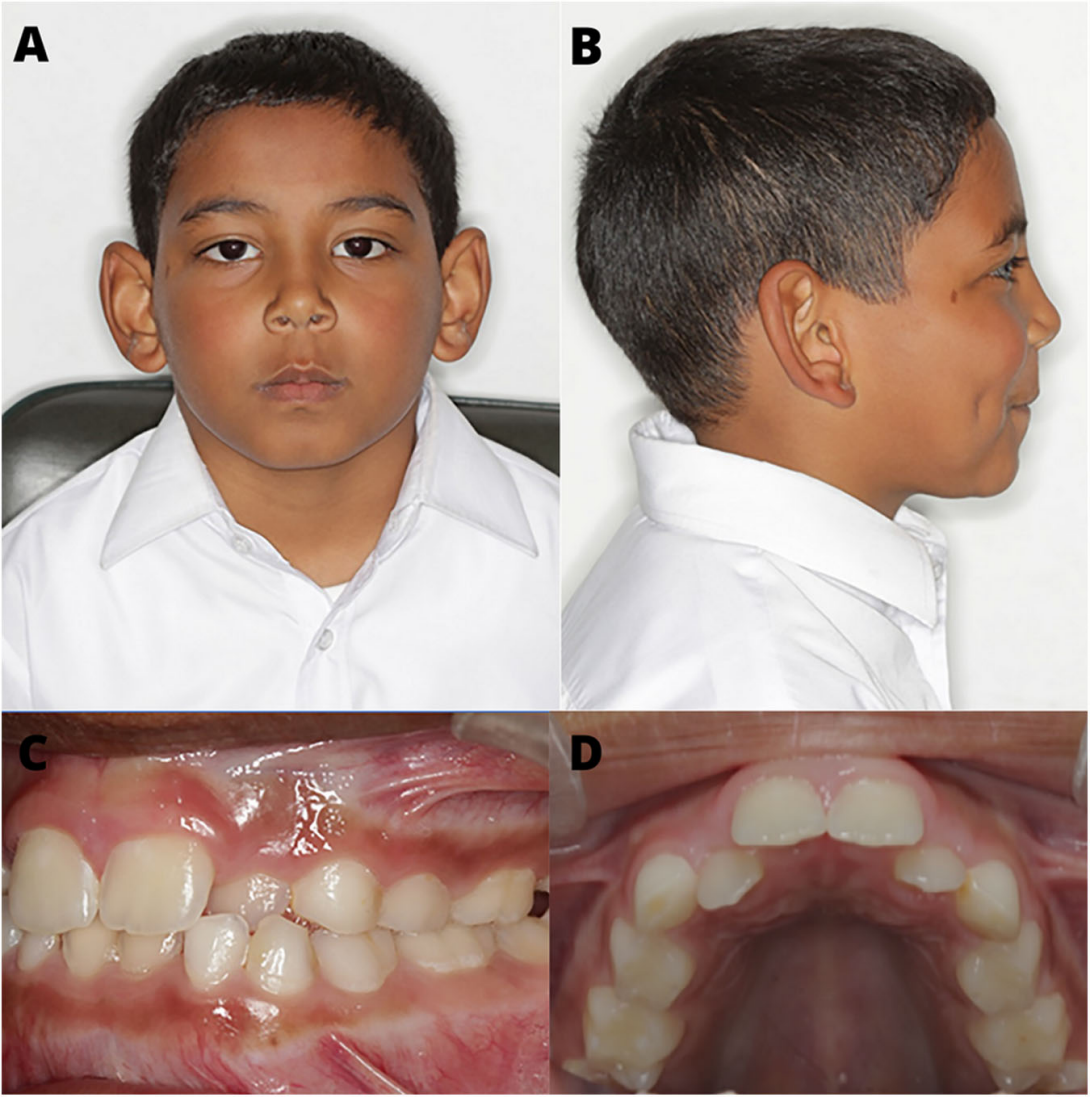
Facial and intra-oral images of the 10-year old male with PD. **A**, **B** Prominent ears and a flat frontal superior third of his face are evident. **C** Maxilla displaying dental crowding and a high-arched palate. **D** An intraoral image of the teeth in occlusion. There is crowding of his upper anterior teeth and a Class III dental occlusion.

The affected individual had an unaffected older sister aged 16 years. His parents indicated no history of consanguinity, but they stated that their third-degree relatives had originated from the same remote geographical region of the Western Cape. There were no links regarding the family’s surnames with the previously described South African patients. The affected boy was of normal intelligence, and his developmental milestones showed no deviations from the norm. Clinically, his height, weight and head circumference were within normal limits. He gave a history of frequent joint pain. His facial features were mildly dysmorphic, with large, prominent ears and a flat frontal upper third of the face, and a flat facial profile (Fig. 2A, B). His sclerae had a blue tinge and the eyes were mildly downturned. His neck appeared to be broad and short.

**Fig. 3:**
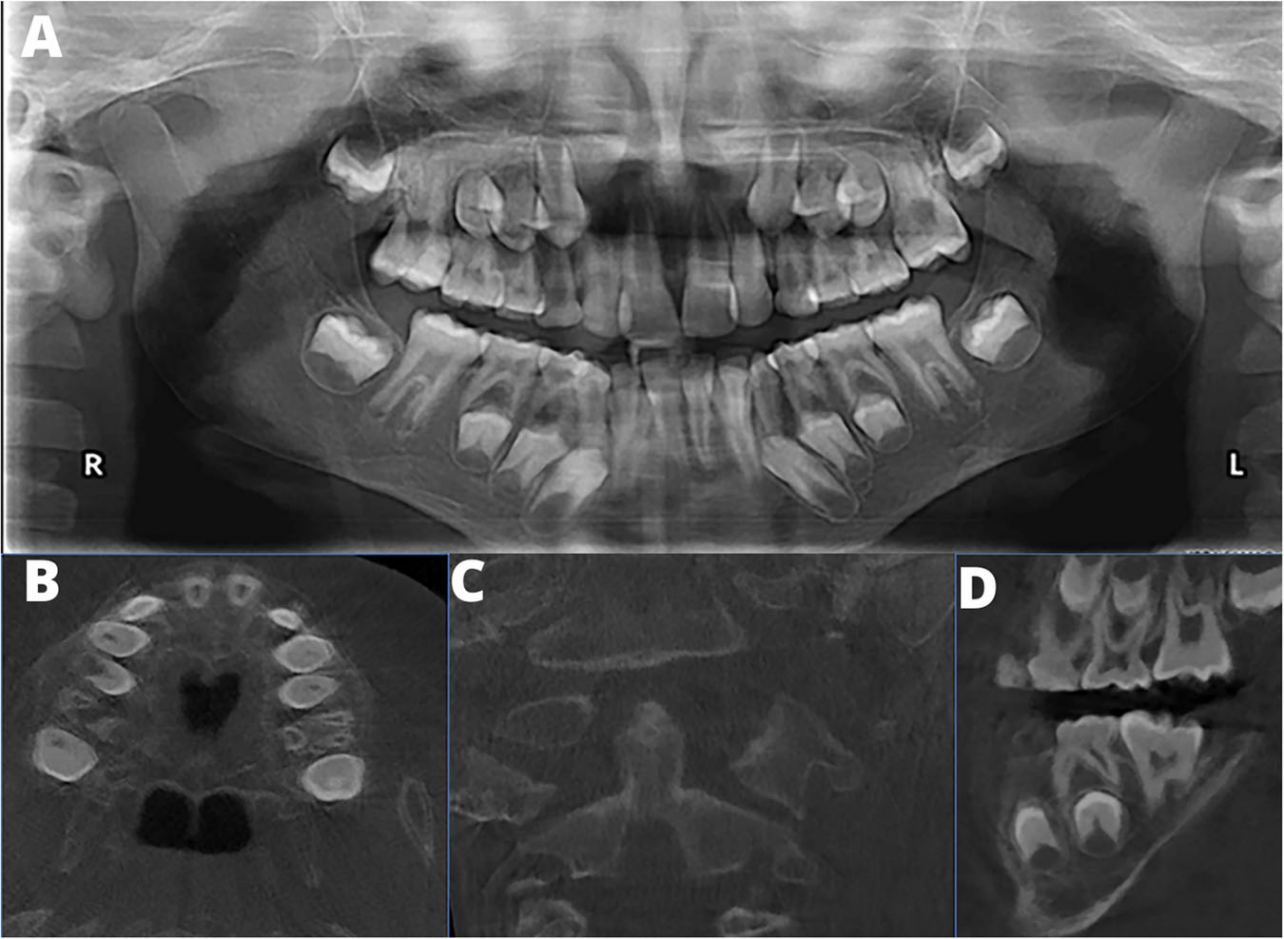
Craniofacial and oral radiographs of a 10-year old male with PD. **A** Panoramic radiograph of the affected boy. There is dental crowding and delayed exfoliation of several primary teeth. Mesotaurodontism of the upper and lower first permanent molars is evident. The mandibular condyles lack a constriction at the neck. **B** High-arched palate. **C** Abnormal shape and decreased density of C2. **D** Mesotaurodontism of his first permanent molars.

An intraoral examination (Fig. 2C, D) revealed the presence of his first permanent molars and his permanent maxillary and mandibular incisors. He had retained mandibular primary incisors that were extracted prior to the photographs being taken. The primary canines and molars were also present. No other permanent teeth were visible. His oral hygiene was satisfactory, and no carious lesions were evident, but a few areas of enamel demineralisation were visible as white-spot lesions. The colour of the dentition appeared to be within normal range, and relatively consistent. There was crowding of his upper anterior dentition, and teeth 12 and 22 were palatally displaced and in cross-bite with the lower lateral incisors. The primary canines showed signs of uneven wear. He also had a high-arched palate (Fig. 2D). There was a band of light-brown pigmentation on the mandibular and maxillary attached gingiva. Due to the dental crowding, panoramic (Fig. 3A) and cephalometric radiographs (Supplementary Fig. 1) were obtained to assess the dentition’s integrity and facilitate the necessary referral to the department of orthodontics.

The panoramic and cephalometric radiographs revealed retained primary mandibular incisors (72 and 82). There was a delay in the eruption and root apexification of the permanent teeth by approximately 3 years. The first permanent molars, especially the 36 and 46, showed features of mesotaurodontism (Fig. 3A, D). The lamina dura was absent and there was generalised rarefaction of the jaw with thinning of the cortical bone. The neck of the mandibular condyle lacked its normal constriction. A cephalometric analysis (Supplementary Fig. 1) indicated a steep cranial base, a skeletal Class III jaw relationship and a dental Class III molar relationship. The mandible and maxilla were retrognathic resulting in a flat facial profile. The sella turcica appeared small but was found to be within normal range; it had a narrow opening but no bridging (Supplementary Fig. 1). The maxillary sinuses appeared relatively opacified on the panoramic radiographic but were clearly visible on the lateral skull and the cephalometric radiographs.

Following an orthodontic evaluation, a cone-beam computed tomography (CBCT) was requested for a comprehensive evaluation of his cervical spine and the formulation of a treatment plan. An expert maxillofacial radiographer assessed the images and noted the following: the CBCT images revealed a high palatal vault (Fig. 3B); There was evidence that his craniofacial bones were undergoing osteoporotic changes, especially in the jaws and cervical vertebrae (Fig. 3C, D); the bone density was visibly decreased, represented by reduced trabeculae and decreased opacity of the bone; the thickness of the cortical bone of the zygomatic arch, maxilla, mandible and vertebral bodies (Fig. 3C) of the boy were found to be relatively thin. The paranasal sinuses were found to be in normal volumetric ranges for his age. Software to quantify these findings was not available.

After the initial visit and radiographs, the retained mandibular primary incisors were extracted. The ultrastructure of the enamel (Supplementary Fig. 2) and dentine was compared to that of a tooth 82 from an unaffected, non-syndromic, sex- and ethnicity-matched 6-year-old from the paediatric clinic whose tooth had exfoliated. Using scanning electron microscopy (SEM), the enamel surface from the child with PD showed some evidence of increased porosity. The significance of this finding is unclear.

The percentage of the various ionic components of the surface of the crown and the root were also measured and compared. These values are tabulated in Supplementary Table 1. There were minor differences in the atomic percentage of the elements between the enamel of the crown and the roots of the unaffected child and the affected child. In particular, the element magnesium was undetectable from the root of the child with PD, compared to 0.3 at.% in the unaffected child. Conversely, Aluminium and Silicon was detected in the root of the child with PD (Al = 0.16 at.%, Si = 0.76 at.%) and were undetectable in the unaffected child’s root.

### Molecular analysis

#### Sanger sequencing

**Fig. 4:**
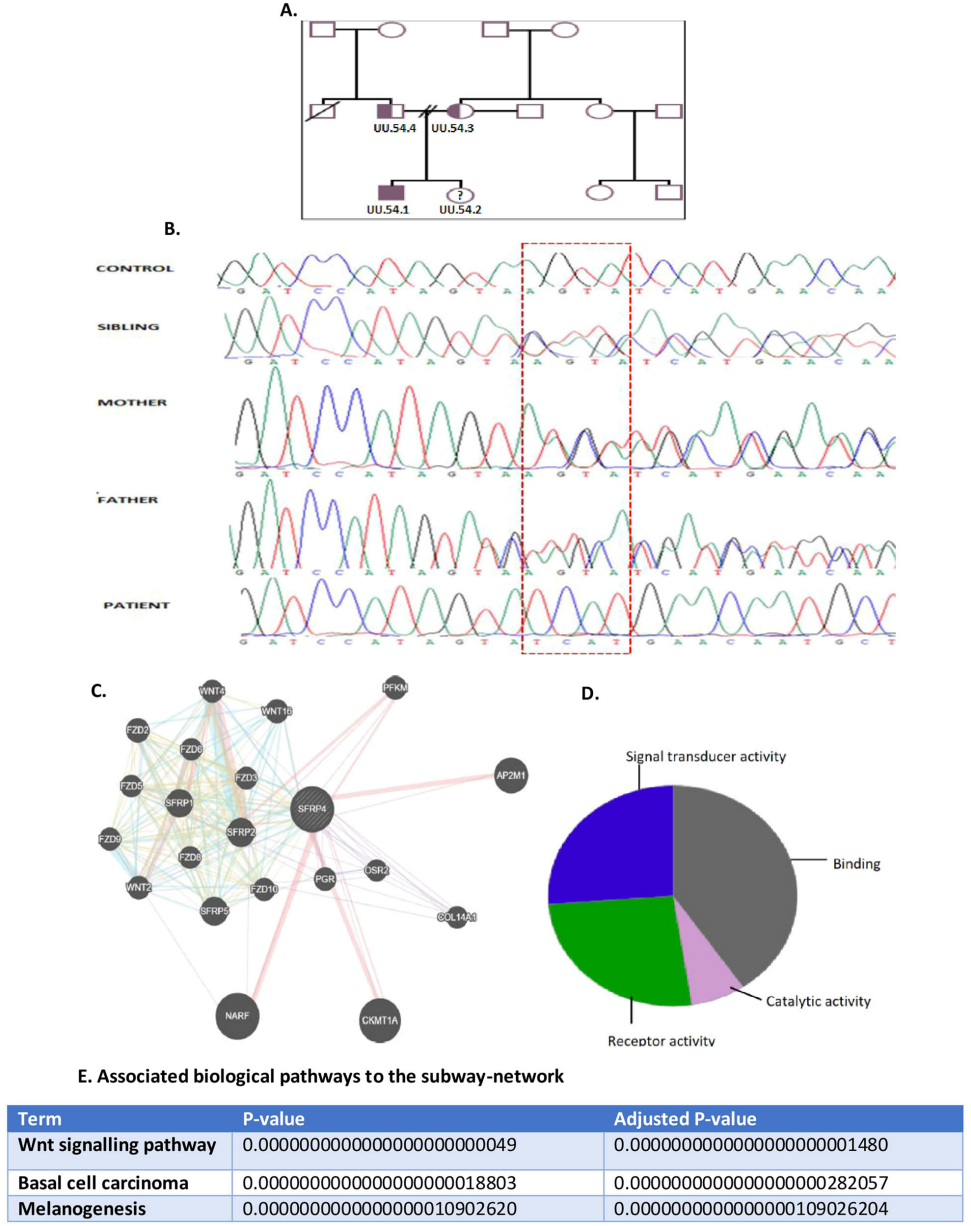
Analysis of molecular findings from the patient with PD. **A** The pedigree of the affected patient, indicated with an arrow and unaffected sibling and non-consanguineous parents (adapted from Wonkam et al., 2016 with permissions). **B** Electropherogram showing the novel SFRP4 variant c.855A > (g.116083_116086delAGTA), found in the south African patient and family. Electropherograms A. represent the laboratory control, B., *C*., and D. are the heterozygous parents and the sibling for the deletion. Electropherograms E. represents the homozygous patient for the four nucleotides (AGTA) deletion indicated with a black arrow. **C** The protein network interaction and molecular function associated with SFRP4. The gene SFRP4 was associated with 20 protein pathways (**A**), **B** indicates the associated biological pathways and the molecular functions associated with the network is seen in **C**.

Ethical approval was obtained by the University of Cape Town Human Research Committee. Following the patient’s clinical evaluation, informed consent and assent were obtained from the patient and unaffected parents and sibling (Fig. 4A). Primers were designed for all the six exons of the *SFRP4* gene to perform bi-directional sequencing. Due to limited patient DNA, the parent (UU.54.3) was initially sequenced. Sanger sequencing revealed a full base pair (AGTA) deletion c.855+4delAGTA in intron 5. The patient was subsequently sequenced with primer set 5, revealing the variant and confirming the homozygosity in the patient. Segregation analysis performed on the remaining family members confirmed the variant to be heterozygous in both parents and the unaffected sibling (Fig. 4A). The variant has not been reported in the literature to be associated with any disease phenotypes. No other variants were found in the coding sequences of the *SFRP4* gene in this family.

### Pathogenicity predictions

#### Variant annotation

In silico mutation tools in ANNOVAR was used to predict whether variants are disease-causing, based on the change in either protein or amino acid of the putative variant. However, it was not suitable for predicting single variants in a single sample. Fortunately, a separate analysis using MutationTaster predicted and confirmed the mutation on our identified variant with a probability of 1, representing a high level of confidence in the prediction. MutationTaster predicted that the protein features might be affected and that the splice site changes. The publicly available allele frequency databases; ExAC [16], 1000Genome [17] and gnomAD [18] was used to determine the allele frequency. The variant in this study was not found in these allele frequency databases and is therefore considered novel.

#### Splice site predictions

The identification of motifs harbouring intronic mutations that may affect splicing regulatory elements (SRE) was tested using Human Splice Finder (HSF) [19]. The splice site prediction neural network (NNSplice) programme combined the prediction scores for splice site mutations for Maximum Entropy (MaxEnt), Splice Site Finder (SSF) and NNSplice [20].The variant identified in the patient was present in the intronic region (Supplementary Table 2) and the donor site was affected by the mutation (Supplementary Table 3). Positions in the sequence for the 5’ intron are labelled as negative and positive for the 3’ intron. The variation expresses the difference between the wild type (WT) (reference) and mutant values. The variation between the WT and mutant is −27.65, which is below the −10% threshold for HSF, it is, therefore, considered that the mutation breaks the splice site.

#### Gene-set enrichment analysis

Enrichment analysis was performed for the *SFRP4* gene, using Enrichr [21, 22] and Panther [23, 24]. The *SFRP4* gene was queried in the protein interaction network, GeneMANIA [25, 26].*SFRP4* was associated with 20 protein pathways (Fig. 4C). An interactive functional associate network was demonstrated between *SFRP4* and the 20 related genes. A network plot was produced from this gene list, which indicated the *SFRP4* hub proteins. The network is associated with the Wnt signalling pathway (Fig. 4D). The molecular function mostly associated with the network is binding, report activity and signal transducer activity (Fig. 3C). Most of the *FZD* are coupled to the beta-catenin pathway. The Wnt, frizzled class receptor proteins (*FZD*) are associated with *SFRP4*. Wnts are encoded by the *Frzd-1*; which are secreted signalling agents that bind to the FZD. Wnt and receptor activation stabilises the beta-catenin protein.

#### Linkage disequilibrium

Linkage disequilibrium (LD) was calculated to identify the association of the different variants present in different populations. LD plots for the four previously published variants [6, 7] and the variant in this study were computed in R studio, a script in R’s genetics package. These variants were run pairwise and then collectively for all the five variants in RStudio. A *p*-value of 0.1573 was obtained and the linkage between the two is most likely due to chance rather than actual relatedness, as the *P*-value is greater than 0.05. The coefficient, R^2^, is 1.9992, which is greater than 1 and therefore, closely related and inherited in a population.

### 3D protein structure prediction for functional characterisation

Molecular dynamic (MD) simulations were conducted to assess the effect of deletion of 4-base pair deletion (AGTA). The tertiary structure of the *SFRP4* wild type (WT) and mutant were generated using I-Tasser homology webserver [27] (Supplementary Fig. 3). All MD simulations were conducted with the GROMACS package, version 4.6.5 [28] using AMBER99SB-IDL force field [29]. The system was simulated in cubic box and solvated in water TIP3P model [30]. The temperature and pressure were maintained at 300 K using the Parrinello–Donadio–Bussi V-rescale thermostat [31]and a pressure of 1 bar using the Berendsen barostat [32]. The short-range non-bonded interactions were modelled using Lennard Jones potentials. The long-range electrostatic interactions were calculated using the particle mesh Ewald (PME) algorithm [33, 34]. The LINCS algorithm was used to constrain all bond lengths [35].Then the velocities were assigned according to the Maxwell-Boltzman distribution at 300 K.

## Discussion

This study presents the molecular, craniofacial and dental features of a 10-year-old South African male of Mixed Ancestry with PD caused by a novel homozygous mutation of c.855A+delAGTA, of the *SFRP4* gene. We also provide the SEM and ionic component analysis of the teeth of the affected individual.

There is a paucity of literature regarding the dental and craniofacial manifestations and management of PD. The most frequently reported dental findings are prognathism, dental caries and malocclusion [2, 5, 14, 15]. The presented patient presented bi-maxillary retrognathism, minor malocclusion, and no dental caries. The severity of his craniofacial and dental features appeared to be relatively mild compared to what was previously reported in the literature [2, 5, 14, 15]. This may be due to phenotypic variability or his young age. He will be followed up and monitored in future.

The affected patient presented with retained deciduous teeth as well as delayed development and eruption of his permanent teeth. The available literature suggests that these findings are not unique to this case [14]. Although the affected patient requires orthodontic treatment for maligned teeth, the prognosis of orthodontic treatment is questionable. The absence of the lamina dura is indicative of a compromise in the integrity of the periodontal ligament. A healthy periodontal ligament and optimal activity of osteoclasts and osteoblasts are necessary for successful orthodontic therapy. Histological examination of a section of bone from the proximal femoral metaphyseal area revealed paucity in the number of osteoclasts [36]. Additionally, there are concerns about bone fragility and the effect of orthodontic forces and trauma on the jaws during extractions and surgical intervention. However, favourable post-osteotomy healing has been reported in a case of PD [37]. Nevertheless, it is recommended that minimum force be used during dental procedures. Atraumatic extraction techniques are recommended. Also, good oral hygiene practices and the avoidance of a cariogenic diet are important as caries and the accelerated development of periodontal disease [38] have been reported. Our patient, however, did not present with significant carious lesions. Further research is required to determine if the increased susceptibility to caries previously reported [14, 15] truly has genetic origins. Additionally, our patient presented with taurodontism, which can prove challenging if endodontics or extractions are required [39]. If the need for general anaesthesia arises, it is important for the anaesthetist to be aware of the abnormality in shape and density of the odontoid process and hence ensure gentle manipulation of the patient’s airways. Documentation and further studies of more cases of PD are recommended to confirm that PD causes rarefaction of the jaws and would help establish a more consistent description of the dental and craniofacial manifestations.

The authors used SEM to analyse the elemental composition of the extracted, retained tooth of the patient to the exfoliated tooth from an unaffected six-year-old. This had several drawbacks; exfoliated teeth generally have very short roots compared to retained teeth which require extractions and, therefore, less tooth material is available for examination. Studies have found that the trace elements in deciduous teeth change with age [40]. The elements of Cr, K, Pb, Cu, Cd, Mg, Ca were all found to decrease with age [41]. Like bone, teeth ionic composition is related to environmental exposure and is better studied in individuals with similar diets and from the same populations. In our study, the control was selected out of convenience. Magnesium was undetectable from the root of the child with PD, compared to 0.3 at.% in the unaffected child; Aluminium and Silicon were detected in the root of the child with PD (Al = 0.16 at.%, Si = 0.76 at.%) and were undetectable in the unaffected child’s root. Although we know that the Wnt signalling pathway, in which the *SRPF4* gene plays a role, is important in tooth formation and eruption [42], we do not yet know the impact the pathogenic *SRPF4* gene variation has on tooth development. Further studies on affected patients and animal models would be needed to confirm whether these findings represent true associations.

In the current study, a novel homozygous mutation has been identified in the fifth intron, c.855A+delAGTA, of the *SFRP4* gene in a South African patient presenting with PD, the first report from a patient in Africa. The variant is segregated in the family according to an expected autosomal recessive pattern of inheritance. In silico mutation tools in ANNOVAR was not suitable for predicting single variants in a single sample, which shows the value of using prediction tools when functional studies are not feasible available or functional data is not available. The variant was identified in the donor site. MutationTaster predicted that the splice site changes and Human Splice Finder interpreted the alteration of the wild type donor site, therefore the variant may cause aberrant splicing. These results are predictions, and the accuracy questionable. However, these in silico splicing site tools confirmed that the four base pair deletion (AGTA) in intron 5 (3 nucleotides after exon 5) was present in the donor site, and it is possible that exon 5 may be omitted in the final protein when it is transcribed. In addition, the variant is not present in any of the publicly available databases, which supports this variant to be disease-causing.

A total of five *SFRP4* mutations are mentioned in this report, four deletions resulting in a frameshift and truncated protein, and one possibly affecting splicing. The five variants observed in the same gene are not in linkage disequilibrium, and as a result, there is no correlation between the variants seen in the different populations. The alleles for the neighbouring variants of each ancestral group are not associated with each other and are, therefore, unlinked.

A study by Simsek et al. (2016), produced a mouse model that manifested the PD phenotype occurring in humans to investigate the mechanisms involved in altered bone architecture. This study provided evidence that mice deficient in *SFRP4* have increased trabecular bone growth and a remarkably thin cortical bone due to differential regulation of the WNT and bone morphogenetic protein (BMP) signalling. The impaired shape of the cortical bone is indicative of a failed cortical bone remodelling process [7]. The in silico protein prediction demonstrated a change in the protein length of the mutated SFRP4 protein, which may impact the protein structure. The most important limitation of this study is that functional data is not available for the identified *SFRP4* c. 855A+delAGTA variant. The functional prediction is that exon 5 may be omitted during transcription. Therefore, functional studies are needed to investigate how it affects RNA expression and how the altered protein functions. Although mutations in the promoter region are not common, it is possible and may affect gene expression. The promoter region of *SFRP4* was not investigated for variants in this study. Deep intronic sequences were also not analysed, revealing additional variants that may play a role.

## Conclusion

This study has reported the implication of a mutation in the *SFRP4* gene in an African patient presenting with PD. The study adds to the evidence that PD is seemingly due to mutation in the single gene in many world populations. Understanding PD molecular pathophysiology may provide insight and a better understanding of bone remodelling and can provide insight into the establishment of treatment through understanding the involvement of genes linked to the *SFRP4* gene hub. *SFRP4* was shown to interact with 20 protein pathways, as indicated in Supplementary Fig. 4. The interactions show significant association with the Wnt signalling pathway. Deviations in Wnt signalling leads to the bone abnormalities characteristic of PD. This extremely rare disease affects the global architecture of bone. In contrast, common bone diseases (osteoporosis and osteogenesis imperfecta) affects the bone nanostructure. Since knowledge on bone at the nanoscale is limited [40], the current study of PD in a South African patient may provide an indication of bone structure and function. Results obtained for rare diseases may provide an insight into more common bone diseases. Despite the rarity of PD, dental practitioners may be the first to note the signs and symptoms of this condition and awareness of PD and other skeletal dysplasias needs to be raised amongst dental practitioners. Prevention of dental caries and periodontal disease is essential in the management of those affected.

## Supplementary information

Supplementary Information
