# Supplementary material for: Craniofacial, dental, and molecular features of Pyle disease in a South African child
Source: BDJ Open. 2022 Sep 22;8:28. doi: 10.1038/s41405-022-00120-w (PMC9500065; doi:10.1038/s41405-022-00120-w)

Supplementary Material

Supplementary Figure 1*: Cephalometric radiograph and Cephalometric tracing of the 10 year old male with Pyle Disease*

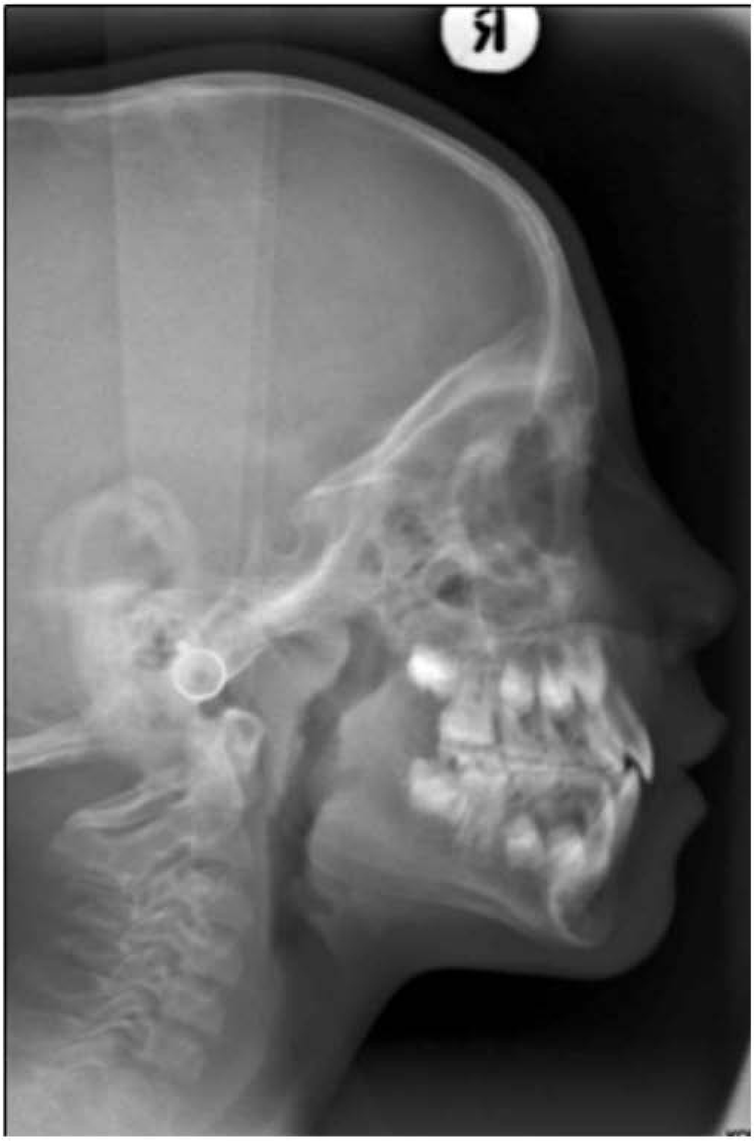

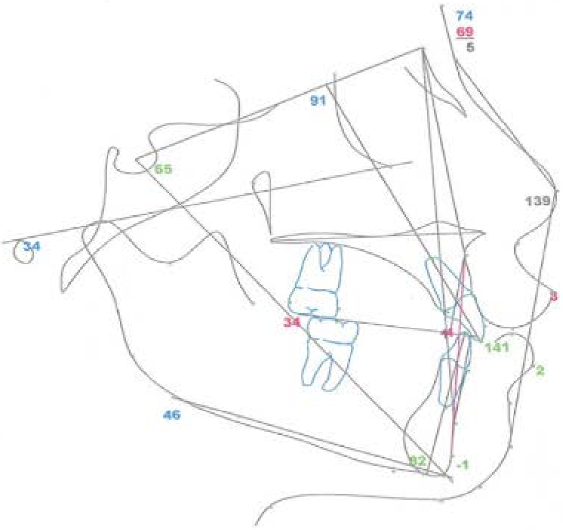

Cephalometric tracing of the affected boy

	Value	Norm	Std Dev	Dev Nor
Interincisal Angle (U1-L1) (°)	141.1	130.0	6.0	1.8 *
IMPA (L1-MP) (°)	82.3	95.0	7.0	-1.8 *
ANB (°)	5.6	1.6	1.5	2.6 **
Lower Lip to E-Plane (mm)	1.8	-2.0	2.0	1.9 *
Upper Lip to E-Plane (mm)	2.9	-3.2	2.0	3.0 ***
MP - SN (°)	45.9	33.0	6.0	2.1 **
SNA (°)	74.1	82.0	3.5	-2.3 **
SNB (°)	68.6	80.9	3.4	-3.6 ***
U1 - SN (°)	90.8	102.4	5.5	-2.1 **
L1 - NB (mm)	4.0	4.0	1.8	0.0
U1 - NA (mm)	-0.5	4.3	2.7	-1.8 *
U1 (labial surface) to NA (mm)	1.0	4.3	2.7	-1.2 *
Pog - NB (mm)	-1.2	1.5	1.7	-1.6 *
Soft Tissue Convexity (°)	139.2	135.9	4.0	0.8
SN - GoGn (°)	44.8	32.0	5.0	2.6 **
Facial Angle (FH-NPo) (°)	79.7	87.2	3.0	-2.5 **
Wits Appraisal (mm)	-4.5	-1.0	1.0	-3.5 ***
FMA (R4 Version) (°)	34.3	25.3	4.5	2.0 **
L1 Protrusion (L1-APo) (mm)	1.2	2.7	1.7	-0.9
S - A (mm)	74.9	89.8	-17%	
Mandibular Length (Jarabak G - Me	54.4	76.2	5.0	-4.4 ****
Maxillary length (ANS-PNS) (mm)	45.7	51.6	4.3	-1.4 *
Y-Axis -- Downs (SGn-FH) (°)	65.2	60.7	3.4	1.3 *
Nasolabial Angle (Col-Sn-UL) (°)	106.4	102.0	8.0	0.6
Overjet (mm)	3.5	2.5	2.5	0.4
Overbite (mm)	1.6	2.5	2.0	-0.5

SUMMARY ANALYSIS

Supplementary Figure 2: SEM digital image of the enamel surface of a tooth from an unaffected child (left) and evidence of surface enamel porosity (right) in the tooth of the individual with Pyle Disease

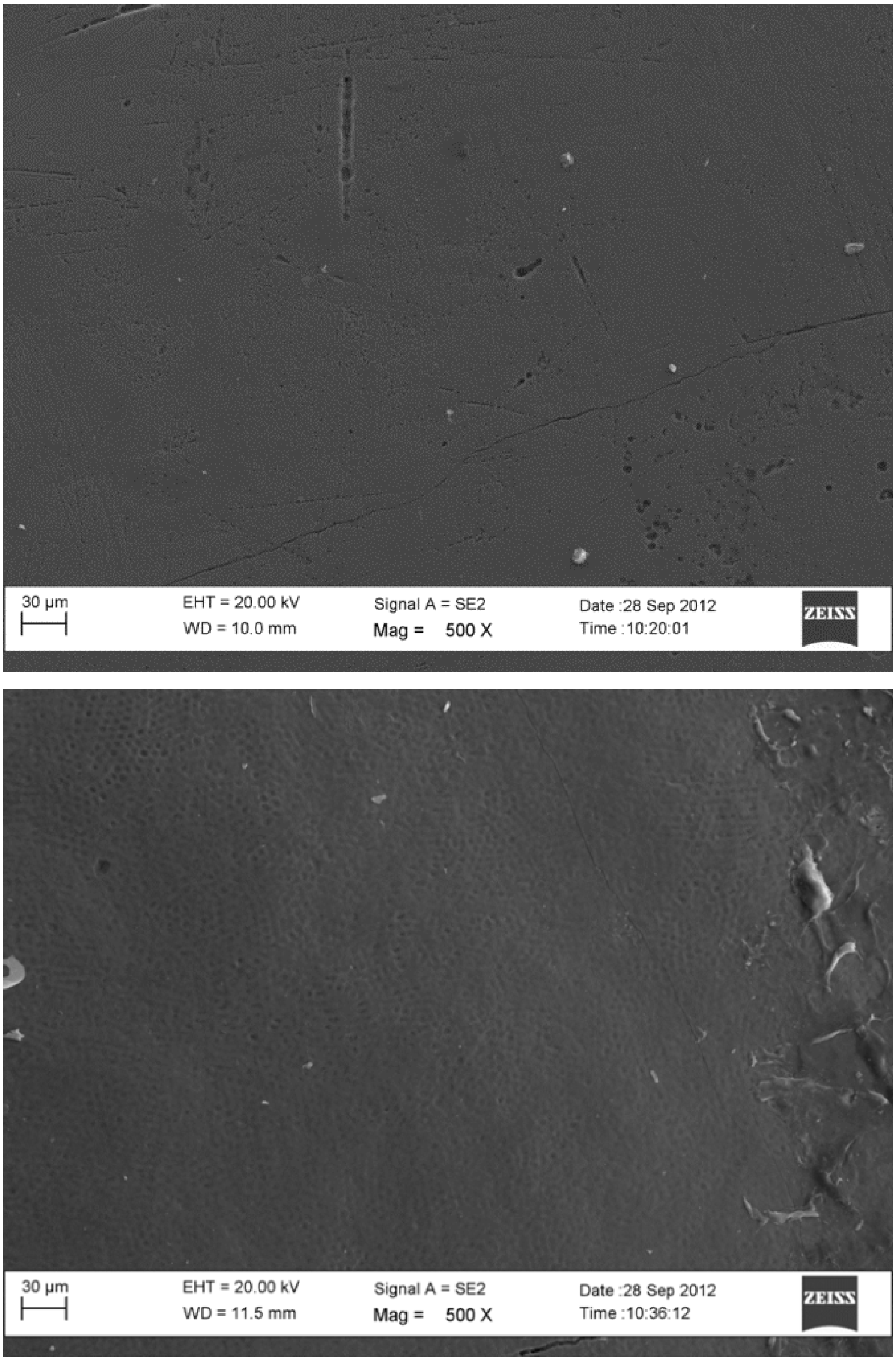

Supplementary Table 1 Ionic components of the tooth surface of an unaffected child compared with the tooth surface of the male with Pyle Disease.

	**Normal Child:**	**Affected Boy:**
**Element**	**Crown Atomic percentage**	**Crown Atomic percentage**
**O**	65.95	68.74
**Na**	0.72	0.78
**Mg**	0.15	-
**P**	13	11.95
**Cl**	0.38	0.32
**Ca**	19.8	18.21
Total	100	100
**Element**	**Root Atomic percentage**	**Root Atomic percentage**
**N**	21.66	19.49
**O**	68.89	73.41
**Na**	0.42	2.64
**Al**	-	0.16
**Si**	-	0.76
**Mg**	0.3	-
**P**	3.52	1.01
**S**	0.52	1.16
**CL**	0.25	0.74
**K**	0.07	0.09
**Ca**	4.37	0.52
**Total**	**100**	**100**

Supplementary Table 2| Representing the Human Splice Finder matrices analysis of the variation between wild type sequence and the mutation (c.855+4delAGTA) in *SFRP4*

Predicted signal	Prediction Algorithm	cDNA Position	Interpretation	WT (%)	Mutant (%)	Variation (%)
**Broken WT Donor site**	HSF Matrices<br>MaxEnt	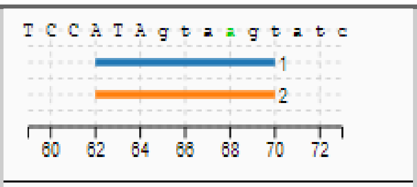	Alteration of the WT donor site, most probably affecting splice	83.72	60.57	-27.65

*WT, wild type

Supplementary Table 3| Representing the splice site analysis by NNSplice and scores obtained for the c.855+4delAGTA in *SFRP4*

Splice signal predictor	Score prediction
**MaxENT**	-100
**NNSplice**	-100
**SSF**	-35.7

*0 represents no effect and -100 indicates that splicing is affected. Position in sequence for the 5' intron is labelled as negative.

Supplementary Figure 3: The quaternary structure of the SFRP4 protein. The predicted 3D structure of c.855+4delAGTA variant and the corresponding WT SFRP4 protein. Note the change in the length of protein, A. from 346 (WT) to B. 379 (c.855+4delAGTA variant).

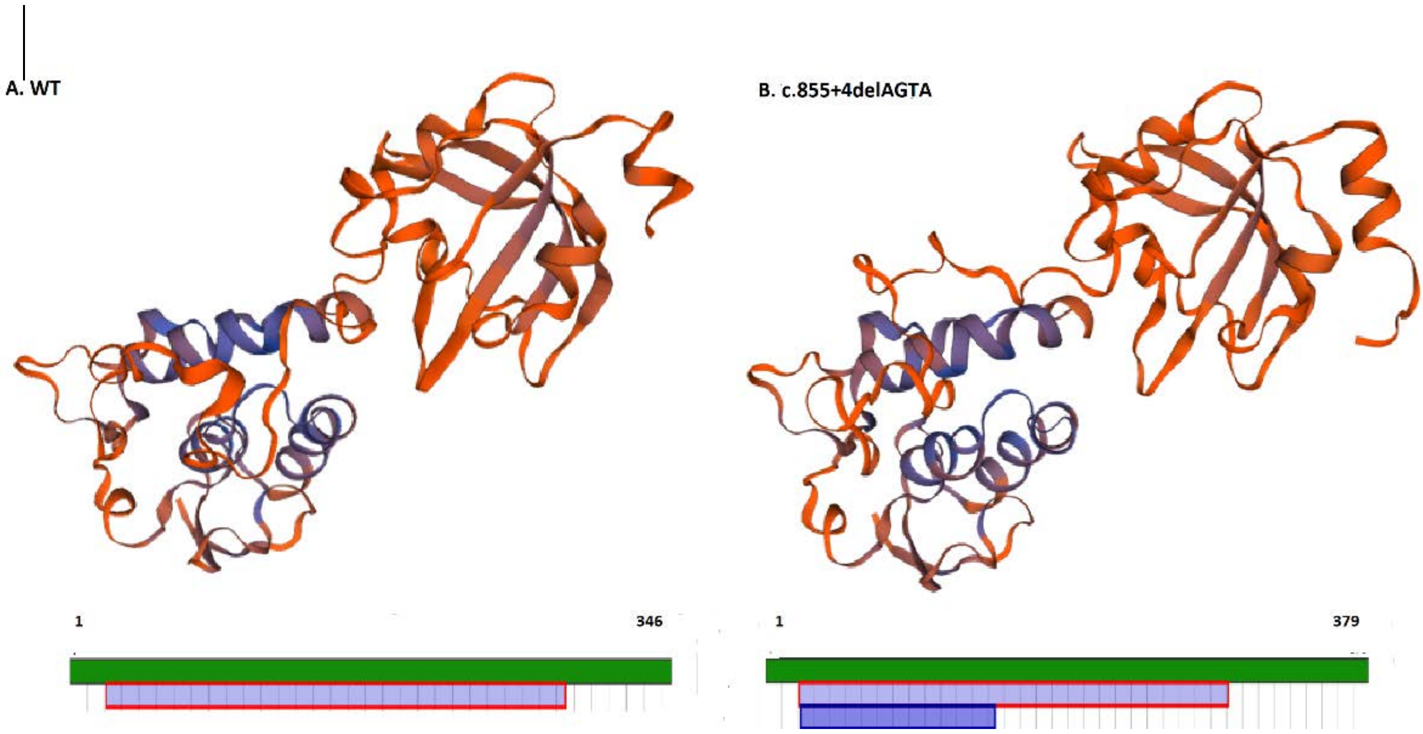

The 3D quaternary protein structure of the SFRP4 Wild-type (WT) and the mutated protein was predicted using the SWISS-MODEL homology-modeling server (Waterhouse et al., 2018). No mouse structure was available, therefore, the frog structure of the *Xenopus laevis* (5xgp.1) was used as a template. This template was selected according to the homology and global model quality estimate (GMQE). A low GMQE was seen in the WT and the mutated protein, 0.39 and 0.34, respectively. The X-ray crystallography was high (2.1Å) for both WT and mutant. The length of the mutated protein (379) is longer than the WT (346), this is due to the presence of the variant being within a spice site and as a result may affect the binding of substrates and the cellular membrane

Supplementary Figure 4: Protein structure prediction for Functional Characterisation of the SFRP4 protein

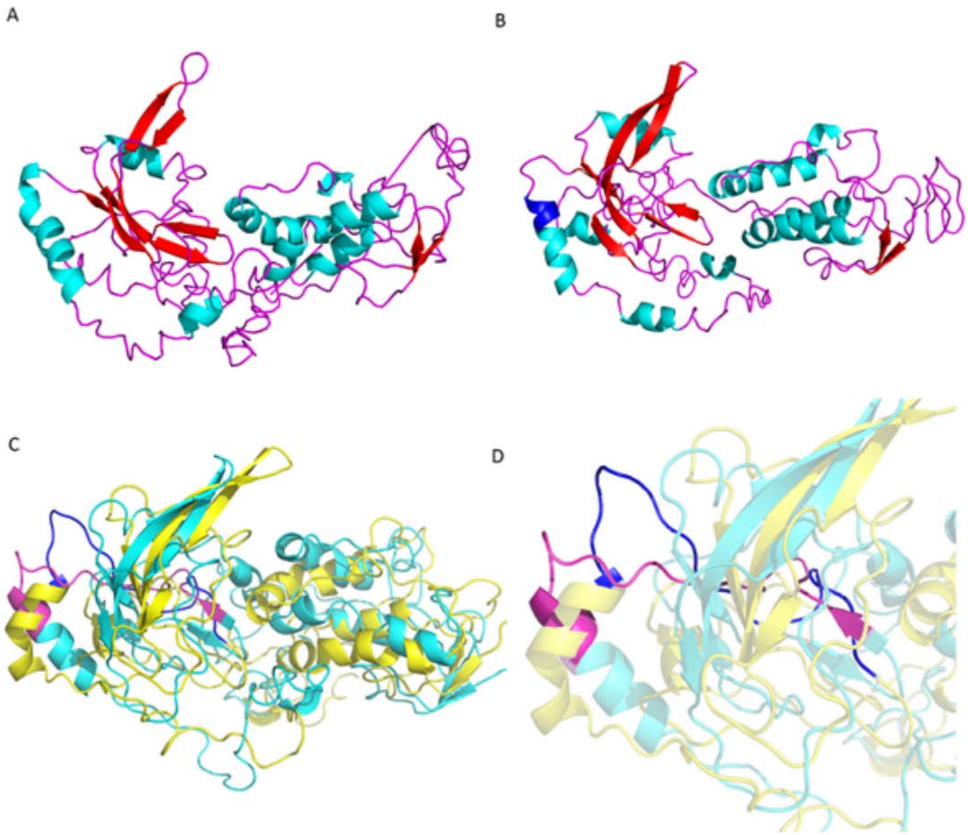

Supplement: Supplementary file 1 — Supplementary Information [file 41405_2022_120_MOESM1_ESM.pdf]
